# pH-Responsive Chitosan-Doped ZnO Hybrid Hydrogels for the Encapsulation of Bioactive Compounds in Aquaculture

**DOI:** 10.3390/polym15204105

**Published:** 2023-10-16

**Authors:** Samuel Sánchez-Serrano, Daniela J. González-Méndez, José A. Olivas-Valdez, Natalie Millán-Aguiñaga, Viridiana Evangelista, Oscar E. Contreras, Marlene N. Cardoza-Contreras

**Affiliations:** 1 Marine Sciences Faculty, Autonomous University of Baja California, Ensenada 22860, Mexico; sanchez.samuel@uabc.edu.mx (S.S.-S.); josaphat.gonzalez@uabc.edu.mx (D.J.G.-M.); olivasja@uabc.edu.mx (J.A.O.-V.); nmillan@uabc.edu.mx (N.M.-A.); vevangelista@uabc.edu.mx (V.E.); 2 Nanosciences and Nanotechnology Center, National Autonomus University of Mexico, Ensenada 22800, Mexico; ocontreras@ens.cnyn.unam.mx

**Keywords:** hybrid hydrogels, chitosan, zinc oxide, antimicrobial, pH responsive

## Abstract

In this study, we synthesized and characterized pH-responsive Chitosan–AgCl-doped ZnO hybrid hydrogels and evaluated their potential for loading aquaculture bioactive compounds, and assessed their antimicrobial properties against a threatening pathogen associated with disease across a broad spectrum of warm water fish and invertebrates. Hydrogel characterization consisted of assessing morphology via SEM, composition via EDS, hydrogels’ network components interactions via FT-IR and pH response through swelling behavior determinations. The swelling characterization of the synthesized hydrogels demonstrated a pH-responsive behavior, showing that low pH values caused the hydrogel polymeric network to expand and capture more of the aqueous solution. These characteristics make the synthesized hydrogels suitable for the encapsulation and controlled release of drugs and bioactive compounds in aquaculture. Chitosan_ZnO hybrid hydrogels showed great antimicrobial activity against *Vibrio harveyi*, even better than that of loaded PB hydrogels. Here, we provide evidence for the potential capacity of Chitosan_ZnO hybrid hydrogels for the preventive and curative treatment of diseases that impact aquaculture animal health and prevent drug resistance by bacteria.

## 1. Introduction

In recent years, aquaculture has developed worldwide [1,2]. However, this increase in production has come with various phenomena such as the increase in the density of crops. These densities are the cause of agglutination, oxygen reduction, and stressful situations in aquaculture organisms, generating the development of certain pathologies, which can lead to disease and malnutrition [3,4]. For this reason, antibiotics are used in aquaculture mainly to prevent and treat bacterial diseases and are commonly administered orally along food. However, the unintentional consumption of antibiotics in food residue by organisms reared in aquaculture has been shown to cause the development of resistance to pathogenic bacteria in both aquaculture stocks and the humans that consume them [5,6,7,8]. Additionally, a high concentration of antibiotics can remain in the water that is ultimately discharged into the ocean, contributing to pollution [9,10,11]. Due to these problems, it is necessary to eliminate infectious agents without propagating antibiotic resistance. An alternative to orally administered antibiotics is the use of hydrogels to encapsulate drugs, which consist of polymeric chains physically or chemically interlinked, generating a porous three-dimensional network with a high capacity to absorb water while not dissolving in an aqueous medium [12]. They have unique characteristics, such as high bio-compatibility, high surface area, and the ability to release drugs over a prolonged period of time in an affected area as a response to a stimulus (pH change, temperature, etc.) [13]. Hydrogels can be used to transport drugs within the bloodstream or can be absorbed orally due to their compatibility with the physiological environment, their ability to load molecules in a polymeric network, and their adaptability [14,15]. Another convenient characteristic of hydrogels is that they can be decorated, modified, and functionalized [16]. Due to this versatility, hydrogels can be hybrids when combined with other nanoparticles (nps) and nanostructures, both organic or inorganic; this characteristic allows properties derived from nps to be exploited while maintaining the integral characteristics of the hydrogel [17,18]. One possible constitutive configuration of a hybrid hydrogel is to be composed of an organic polymer as the main structure of the network and inorganic nps of an element or compound in a minor proportion. Therefore, chitosan is an excellent option to use as polymeric organic material to form hydrogels, due to its nature and desirable properties such as biodegradability and biocompatibility (it does not present side effects or toxicity when interacting with biological agents) [19,20]. Furthermore, chitosan also appears to have bacteriostatic and mucoadhesive properties [21]. Recent studies demonstrated that incorporating chitosan into the diets of aquaculture organisms tends to improve their growth and weight, in addition to raising their content of polyunsaturated fatty acids [22]. Among the possible nanomaterials that can hybridize a hydrogel for feeding purposes, zinc oxide (ZnO) is an excellent candidate since it is considered a harmless substance [23]. For example, in an acidic environment such as in the stomach, ZnO can act as the most viable source of soluble species of Zn, a well-known nutrient required for normal growth, development, and functioning of all animal species [24]. The main function of Zn is as a cofactor in various enzyme systems and as a component of various metalloenzymes. In addition to what has already been mentioned, ZnO has properties that can enhance the hydrogel’s functionality. For example, it has been shown to have antibacterial, anticancer, antioxidant, and anti-inflammatory properties [25,26,27,28,29]. Another favorable point is that ZnO can be doped (the incorporation of a low concentration of impurities in a semiconductor material) with other materials to enhance its properties [30,31]. Silver is an additional possible dopant for ZnO and an element that has shown antimicrobial, antiseptic, antifungal, and antiviral characteristics [32,33,34]. In this sense, ZnO nanostructures can be doped with elements with strong and proven antimicrobial activity, such as Ag and Cl, to improve its antimicrobial potential [35,36,37]. In addition, we recently [38] found that simultaneously incorporating small concentrations of elements with antimicrobial properties into the ZnO crystal lattice can greatly enhance its effectiveness against pathogenic microorganisms in aquaculture such as *Vibrio* spp.

Alternatively, so-called bioactive compounds include both nutrients and non-nutrients present in the food matrix (vegetal and animal sources) that can produce physiological effects beyond their classical nutritional properties [39]. In aquaculture, bioactive compounds are increasingly used as alternatives to treatments for viral and bacterial infections [40,41]. One of the most studied bioactive compounds for the improvement of animal health of aquaculture species include phytobiotics, which are plant- and algae-extracted compounds shown to have antistress, anti-inflammatory, antimicrobial, antioxidant, and immunostimulatory properties that can also promote the growth and general health of organisms [42,43,44,45,46]. Nevertheless, substituting antibiotics with phytobiotics does not completely solve the core issue since the dosing mode is carried out in the same haphazard way as that of antibiotics, where it is administered together with food. While this may induce some bacterial resistance, it still continues to cause water contamination and problematically, as most phytobiotics do not reach the antimicrobial effectiveness of antibiotics [47]. Since phytobiotics are extracts from various plants, they tend to interact with food compounds and produce odors and flavors that are unattractive to livestock. An alternative to this issue could be the use of hybrid hydrogels with enhanced antimicrobial properties that are able to encapsulate, transport, and release bioactive compounds in aquaculture crops. In this work, we synthesized and characterized chitosan-AgCl-doped ZnO hybrid hydrogels, evaluated their potential for loading and releasing bioactive compounds in aquaculture, and assessed the possible potentiation of their antimicrobial properties against the pathogen *Vibrio harveyi*, associated with disease across a broad spectrum of warm water fish and invertebrates.

## 2. Materials and Methods

### 2.1. AgCl-Doped ZnO Synthesis

First, we obtained a colloidal solution of ZnO nps as carried out previously [39]. Subsequently, AgCl-doped ZnO was synthesized with methods reported in previous work but with some modifications [38]. Here, polyethylene (PE) substrates were immersed in a 1% 1-dodecanethiol (Sigma-Aldrich, Burlington, MA, USA, CH_3_(CH_2_)_11_SH)) solution in methanol (ACS reagent 99.8%, Sigma-Aldrich); then, this solution with PE substrates was put in a 60 °C water bath for 15 min. Subsequently, the PE substrates were removed from the dodecanethiol solution and immersed in the colloidal solution of ZnO nps previously prepared for one minute and then removed from the nps solution. The solvent was evaporated from the substrates via direct heating on watch glass on a hot plate at 60 °C for 10 min. Next, we prepared a 3 mM equimolar precursor solution of zinc nitrate hexahydrate (reagent grade 98%, Zn(NO_3_)_2_·6H_2_O), Sigma Aldrich) and hexamethylenetetramine (ACS reagent 99.8%, C_6_H_12_N_4,_ Sigma-Aldrich) in water. Finally, to achieve ZnO doping, silver nitrate at 2 at. % (2 atoms of Ag for every 100 Zn atoms) and 0.1 N HCl (enough to reach a pH of 5.5) were added to the precursor solution under constant stirring. Once the precursor solution was ready, PE substrates with nps were immersed in it. Finally, the precursor solution with the PE substrates was subjected to a microwave treatment for 60 min at 300 W. The samples obtained were rinsed with distilled water and dried at room temperature. The AgCl co-doped ZnO material grown on the PE substrates was collected with a spatula for future experiments.

### 2.2. Hydrogels Synthesis

#### 2.2.1. Chitosan Hydrogels

First, a solution with 0.2 g of chitosan (from shrimp shells, >75% deacetylated, Sigma-Aldrich) in 20 mL of distilled water was prepared. After, 2% (*v*/*v*) of glacial acetic acid (ACS reagent, ≥99.7%, CH_3_COOH) in distilled water was added to the chitosan solution with constant stirring until a homogeneous and viscous solution was obtained. Moreover, 0.2% (wt./v) sodium tripolyphosphate (Na_5_P_3_O_10_, Sigma-Aldrich) (TPP) aqueous solution was prepared and pH was adjusted to 3 with HCl 1.0 N. This solution was poured into the viscous chitosan solution until an opalescent coloration was obtained; this hydrogel sample was labeled as chitosan. Using a syringe, drops of the last solution with similar volume and weight (8 ± 0.2 g) were deposited on silicon wafers and allowed to stand for 24 h.

#### 2.2.2. Chitosan_ZnO Hybrid Hydrogels

Hybrid hydrogels were prepared following the same procedure described previously for chitosan hydrogels, except that ZnO nanostructures were incorporated into the chitosan solution. An aqueous solution of AgCl co-doped ZnO nanoparticles at 5 wt.% concerning chitosan weight was added to the chitosan solution, before the acetic acid solution, referred to as Chitosan_ZnO.

### 2.3. Hydrogel Characterization

#### 2.3.1. Morphology and Composition

The synthesized hydrogel surface morphology was characterized via scanning electron microscopy (SEM) using a JEOL-JIB 4500 microscope (Tokyo, Japan). Prior to SEM observation, the hydrogel drop samples on silicon wafers were coated with an Au/Pd thin layer in a vacuum evaporation chamber. The elemental analysis was carried out in situ using an energy dispersive X-ray (EDS) microanalysis (OXFORD INCA Energy System, Oxfordshire, England) at an electron accelerating voltage of 15 kV.

#### 2.3.2. Fourier Transformed Infrared (FT-IR) Spectroscopy

FT-IR spectra of liquid hydrogel samples were recorded in a Bruker Tensor 27 Spectrometer (Billerica, MA, USA), in a wave number range from 4000 to 400 cm^−1^. For this, 20 uL of hydrogels samples were put on a KBr plate and sandwiched under another KBr plate to spread the liquid thin layer between the plates. The spectrum of a clean KBr plate (without hydrogel sample) was used for subtraction (background spectrum).

#### 2.3.3. Swelling Behavior

To evaluate how much solution could be loaded into the synthesized hydrogels, a series of 5 aqueous solutions with adjusted pH values of 2, 4, 6, 7, and 8 (with NaOH and HCl) was prepared. Subsequently, an amount of each synthesized hydrogel was weighed in triplicate and each portion was placed in one of the previously prepared aqueous solutions with a different pH. Then, each hydrogel was removed from the aqueous solutions and weighed (excess water on the surface of the hydrogel was previously removed with wipes) every 10 min for 30 min. Finally, the swelling percentage was determined using the following expression:

Swelling % = ((W − W_0_)/W_0_) × 100 (1)

where W is the hydrogel weight after a certain time and W_0_ is the initial hydrogel weight. The statistical analysis to evaluate the loading capacity of the hydrogels consisted of a design relying on two factors (hydrogel composition and pH) and was applied with 2 and 5 levels, respectively, in which the dependent variable, the swelling percentage, was evaluated in triplicate. IBM SPSS Statistics 27.0.0 versión software was used to carry out the analysis and consisted of a two-way ANOVA with α = 0.05, where the assumptions of normality and homoscedasticity were corroborated by the Shapiro–Wilk and Levene tests, respectively. To verify the statistical differences between the pH levels, Tukey’s multiple comparison test was used with a significance of 0.05.

### 2.4. Phytobiotic Hydrogel Loading

The phytobiotic (PB) products used were from Alivira-Karizoo Laboratories (Barcelona, España), Licorol (Lc), whose main components were menthol (89 g/mL) and eucalyptol (92.2 g/mL); the other product used was Coxsan (Cx), composed mainly of carvacrol, thymol, and allicin. Aqueous solutions of each PB products were prepared in a 1:1000 (*v*/*v*). New hydrogels, now with PB loads, were prepared as previously described. Each PB aqueous solution together with the ZnO solution was poured into the chitosan solution right before the acetic acid solution. Finally, this produced Cx- and Lc PB-loaded hydrogel samples, which were labeled as Chitosan_ZnO_Cx and Chitosan_ZnO_Lc, respectively.

### 2.5. Hydrogel Antimicrobial Properties Evaluation

To evaluate the antimicrobial properties of the synthesized hydrogels, the minimum inhibitory concentration (MIC) and its antimicrobial activity against *Vibrio harveyi* were carried out. The *Vibrio harveyi* strain was isolated from an aquaculture fish after a mortality event at an aquaculture farm located in Baja California, Mexico. The isolated strain was cultured in Mueller Hinton liquid media for further DNA extraction. Extracted DNA was used to amplify the 16S rRNA gene. The expected band of approximately 1400 bp was sent to ETON Bioscience Inc. of San Diego, California, for sequencing using the Sanger sequencing platform. EZTAXON database (https://www.ezbiocloud.net/ accessed on 7 July 2022) was used to assign the highest percentage of similarity among type strains.

#### 2.5.1. Antimicrobial Activity of the Synthesized Hydrogels

To determine the antimicrobial activity of the hydrogels, a plate with Mueller–Hinton solid medium was inoculated with the *Vibrio harveyi* strain (1 × 10^8^ CFU/mL). A 10 µL aliquot of each hydrogel was placed (in triplicate) and incubated at 35 °C for 24 h to observe growth. For each treatment, the inhibition halo diameters were measured, in millimeters.

#### 2.5.2. Minimum Inhibitory Concentration (MIC)

To determine the lowest concentration of hydrogels that produced an absence of growth in plates after 24 h of incubation, the standard microbiological tube dilution method was performed. Serial dilutions of the hydrogels were made (with concentrations ranging from 200 to 0.39 µg/mL of each hydrogel) in Eppendorf tubes with 200 µL of Mueller Hinton liquid culture medium (MH). Once the hydrogel dilutions were prepared, 200 µL of *Vibrio harveyi* (1 × 10^8^ CFU/mL) was added into each tube and then incubated at 35 °C for 24 h. After incubation, the tubes were visually examined to identify the first tube with turbidity. Then, 100 µL was taken and inoculated into plates with Muller–Hinton medium from the three tubes prior to the tube in which turbidity was identified. The plates were incubated at 35 °C for 24 h. After incubating, the number of CFU (Colony Forming Units) from each plate were counted in order to determine the MIC of each hydrogel. Growth media, bacterial culture, and antibiotic were used as a positive control, while growth media and bacterial culture were used as the negative control.

## 3. Results and Discussion

### 3.1. Morphology and Composition of Hydrogels

**Figure 1:**
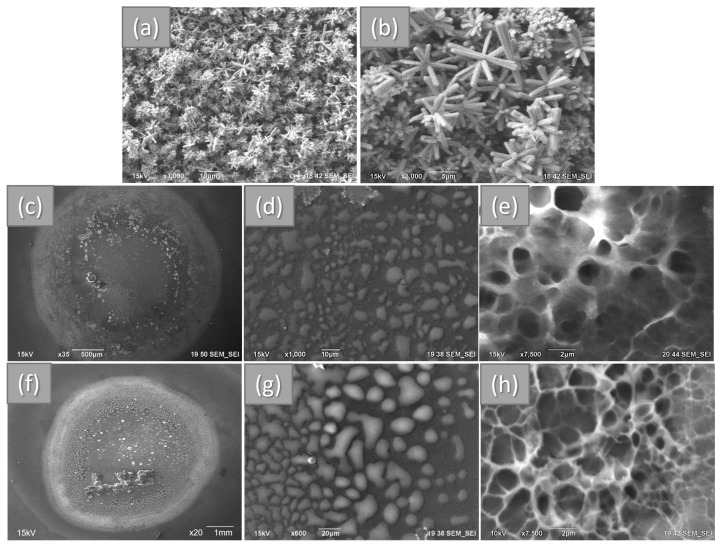
SEM images showing: AgCl-doped ZnO nano and micro rods (**a**,**b**); a whole droplet and an amplified portion of Chitosan (**c**,**d**) and Chitosan_ZnO hydrogels (**f**,**g**); cross section of Chitosan (**e**) and Chitosan_ZnO hydrogel (**h**).

The morphology of the AgCl-doped ZnO samples was expected as follows; arrangements of pin shape clusters formed of rod structures with diameters from 300 nm to 1.0 µm (Figure 1a,b). In the case of Chitosan and Chitosan ZnO hydrogel samples, both consisted of hemispherical-like microdroplets with irregular section shape all over the Si wafer (Figure 1c–f), the dimension of micro-droplet structures is in the interval of 2 to 20 µm. From the cross-sectional SEM images of the hydrogels, a greater porosity and uniformity are observed in Chitosan_ZnO hydrogel than in chitosan hydrogel with pores size in the interval of 0.5 to 1.5 µm.

**Figure 2:**
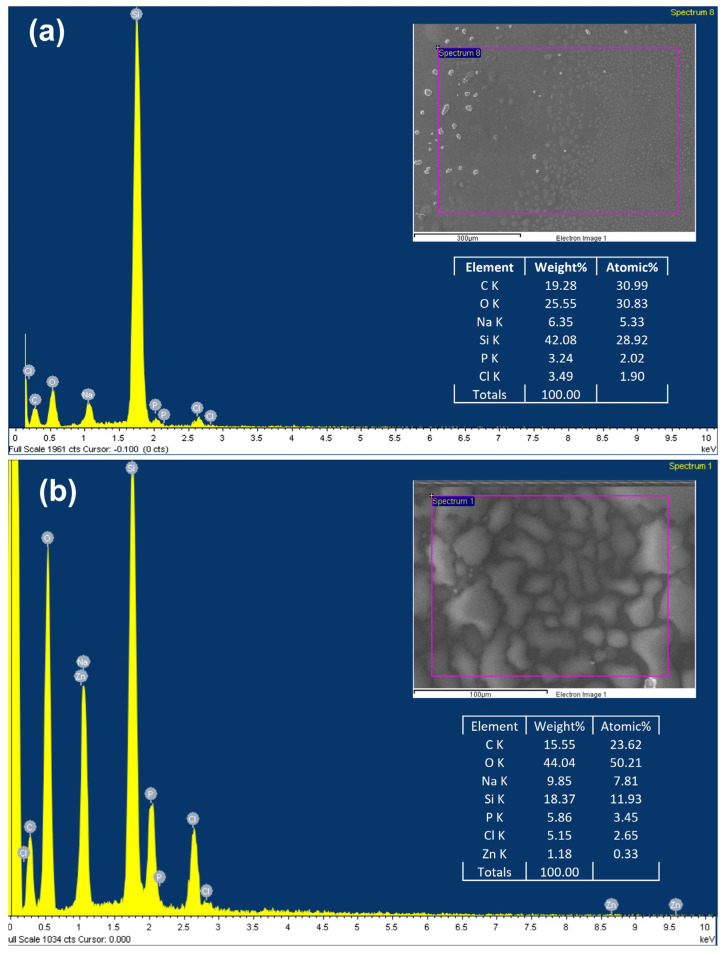
EDS spectrum and chemical composition of (**a**) Chitosan hydrogel and (**b**) Chitosan_ZnO (5 at. %) hydrogel.

Regarding the chemical composition analysis of the synthesized hydrogels, Zn, O, C, and some other elements derived from their synthesis were detected in the EDS analysis (Figure 2). Discarding the Si content (substrate), Zn at. % in the Chitosan_ZnO hydrogels resulted in approximately 0.37 at. % according to the total atoms in the hydrogel, and a 1.38 at. % with respect to C from chitosan. In the same way, Chitosan_ZnO hydrogel showed an increase in oxygen content derived from the ZnO NRs in the composition of the hydrogel.

### 3.2. FT—IR

**Figure 3:**
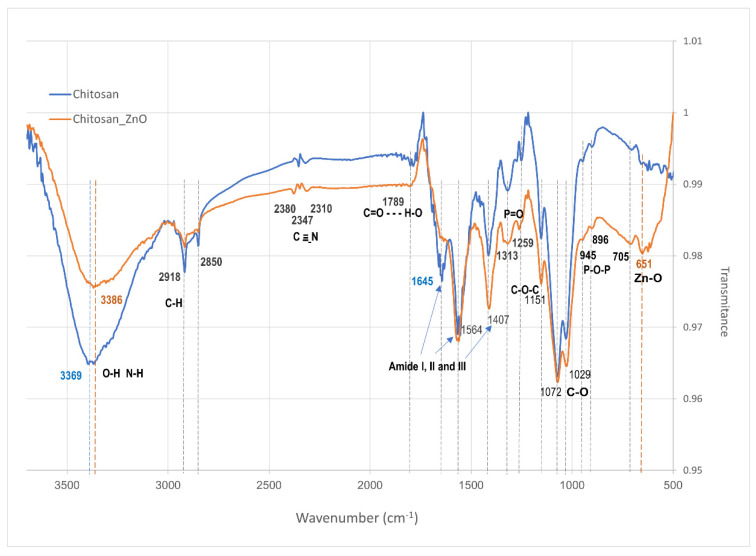
FT—IR spectrums of Chitosan and Chitosan_ZnO hybrid hydrogels.

**Figure 4:**
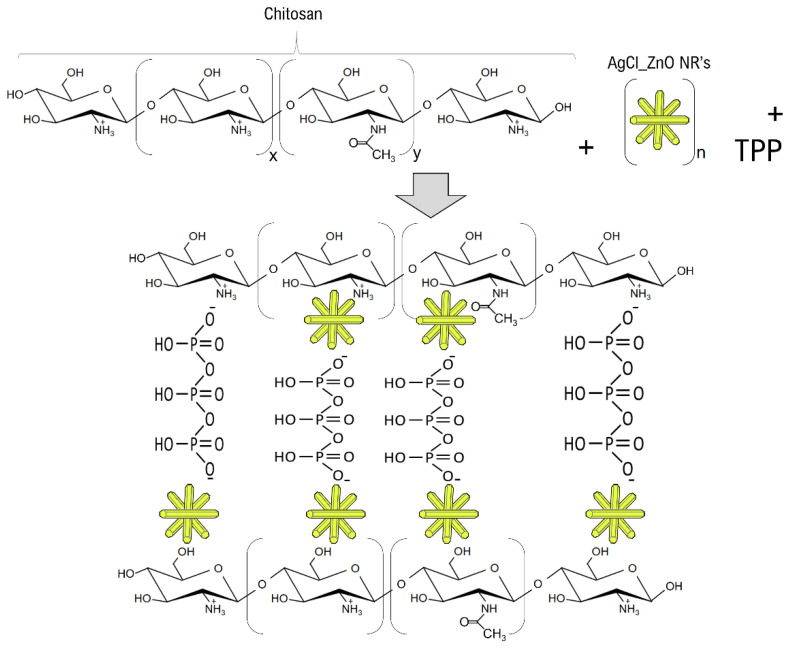
Schematic representation of the Chitosan_ZnO hybrid hydrogel network.

The chemical interactions between the components of the synthesized hydrogels were analyzed via FT-IR (Figure 3). The Chitosan hydrogel and the Chitosan_ZnO hydrogel presented similar absorption bands. Both spectrums show a broad band in the 3324–3045 cm^−1^ region, attributed to the O-H and N-H groups stretching vibrations [48]. The Chitosan_ZnO hydrogel presents a slight shift towards lower wavenumbers (less frequency) implying an interaction of the O-H and N-H chitosan groups with ZnO NRs. The absorption band of both hydrogels present in the region of 2918 and 2850 cm^−1^ correspond to stretching vibrations of aliphatic groups (-CH_2_ and -CH_3_) of the chitosan pyranose ring [49]. A weak band also appears at 2400–2270 cm^−1^ which corresponds to C=N (nitrile) stretching vibrations [50]. TPP phosphate group acts as crosslinker in the synthesized hydrogels causing an absorption band (centered at 1789) which is near amide I region to appear in both spectrums. This could be attributed to hydrogen-bond formation between the chitosan C=O amide group and OH group from TPP phosphate, respectively. In the same way, the P=O stretching vibration attributed to the interaction between the phosphoric anion of the phosphate group and the amino ion from chitosan was found centered at 1259 cm^−1^ [51]. Additionally, absorption bands at 945 and 896 cm^−1^ were attributed to O-P-O asymmetric stretching of TPP phosphate groups [52]. Both hydrogels presented the asymmetric stretching band of C-O-C at approximately 1151 cm^−1^ [53]. The 1072 and 1029 cm^−1^ bands are related to C-O bonds in cyclic compounds like glucose [54,55]. Centered at 651 cm^−1^ an absorption band corresponding to asymmetric Zn–O vibration appeared [56]. The bands at 1645 cm^−1^, 1564 cm^−1^ and 1407 cm^−1^ are attributed to the amide I (C=O stretching), amide II (N-H bending) and amide III (C-N stretching) bonds vibrations, respectively [57,58,59,60,61], while in Chitosan_ZnO hydrogel the amide I band does not appear. Amide I band C=O stretching is hydrogen bonded to N–H of the neighboring intra-sheet chain in the Chitosan_hydrogel [57,58]. The disappearance of this amide band on Chitosan_ZnO hydrogel could be attributed to a new bond formed between ZnO NRs and the chitosan amine and carbonyl groups. According to these results, the Chitosan_ZnO hybrid hydrogel polymer network is formed not only via TPP crosslinking (electrostatic attractions between the ionized amino group of chitosan and the phosphate ion of TPP) [62] but also through interactions of these chemical groups with the ZnO NRs. Based on these results, a schematic representation of the components interactions in the hybrid Chitosan_ZnO hydrogel network is presented (Figure 4).

### 3.3. Swelling Behavior

**Figure 5:**
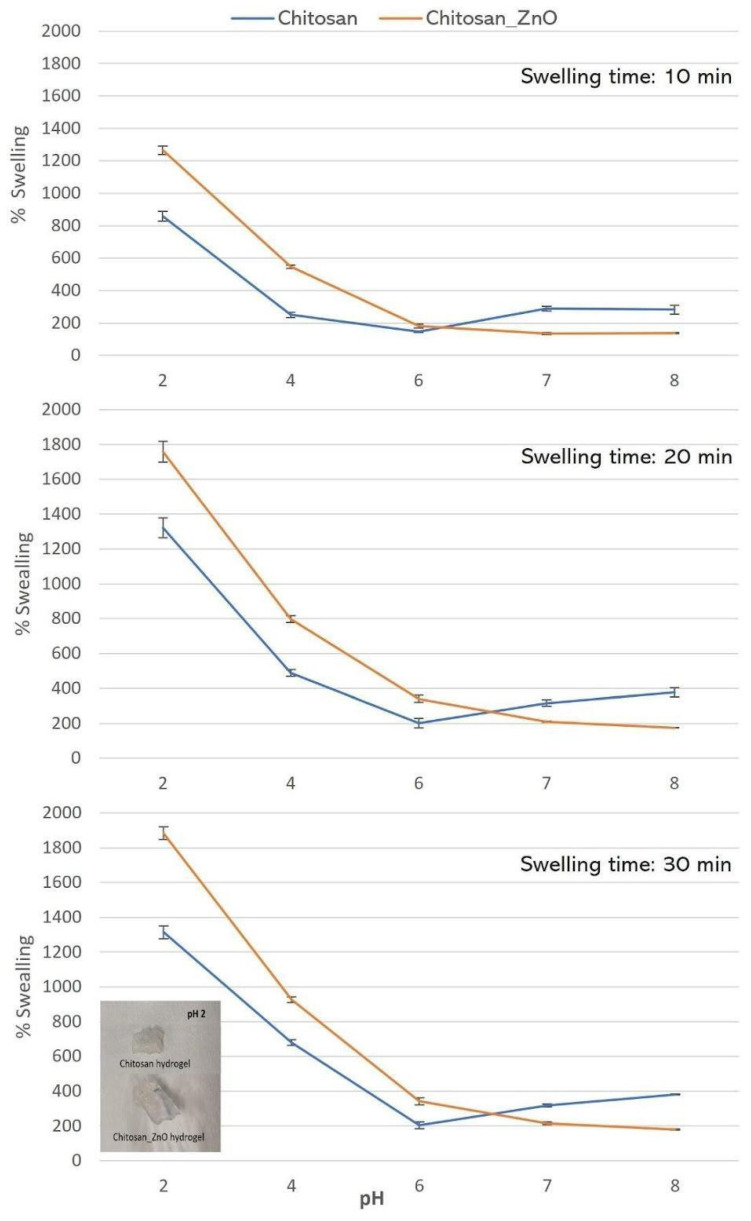
Swelling % of the synthesized Chitosan and Chitosan_ZnO hydrogels after 10, 20, and 30 min of being immersed in aqueous solutions of different pH values. Bottom left inset: Chitosan and Chitosan_ZnO hydrogels swollen after being immersed 30 min in a pH 2 solution.

The swelling behavior of hydrogels provided an overview of the strength and capacity of the polymeric network formed by the components of the hydrogel. The hydrogen proton concentration of the medium in which the hydrogel is located can determine the ionization of various functional groups that make up the polymeric network, affecting the hydrogel’s properties. According to the swelling behavior results (Figure 3), a significant difference was found between the means of the swelling percentage at the different pH levels (*p* < 0.05) for both hydrogel compositions, highlighting that both the Chitosan and Chitosan_ZnO hydrogel had better swelling capacity in acidic solutions. Multiple comparison tests following the Tukey method found that the swelling percentage at pH values of 6, 7 and 8 were not statistically different (*p* > 0.05). The polymeric network of the chitosan hydrogel using TPP as a crosslinking agent can be formed due to the physical crosslinking through hydrogen bonding and dipole–dipole interactions between neighboring ester groups and chitosan chains [63]. However, in acidic aqueous solutions, the repulsion between adjacent ionized residual amino groups (-N^+^H_3_) of the chitosan hydrogel causes the polymeric network to expand, which allows the hydrogel to capture more water [64]. This is desirable since, based on the pH of the stomach (1–6) and intestine (6.5–8.5) of the fish [65,66], loaded hydrogels must be able to withstand pH values below 4 to prevent the disintegration and rapid release of the encapsulated compound in the stomach, and start dissolving at pH values greater than 5, ultimately being solubilized at a pH value near 7. The degradation of the chitosan hydrogels will be greater since, in addition to the pH of the medium, the enzymes present are capable of degrading chitosan (carbohydrases) in the intestine [67]. Figure 5 demonstrates that the Chitosan_ZnO hydrogel has a greater swelling capacity than the chitosan hydrogel in acidic aqueous solutions (6, 4, and 2). This indicates that incorporating AgCl co-doped ZnO in the synthesis of the Chitosan polymeric network increases the swelling capacity of chitosan hydrogels. This is consistent with the observed major porosity of the hybrid hydrogel and with FT—IR results, where possible interactions between chitosan functional groups, TPP phosphoric ions and ZnO NRs could form a bigger network with more interactions and a better swelling behavior.

### 3.4. Minimum Inhibitory Concentration (MIC)

**Figure 6:**
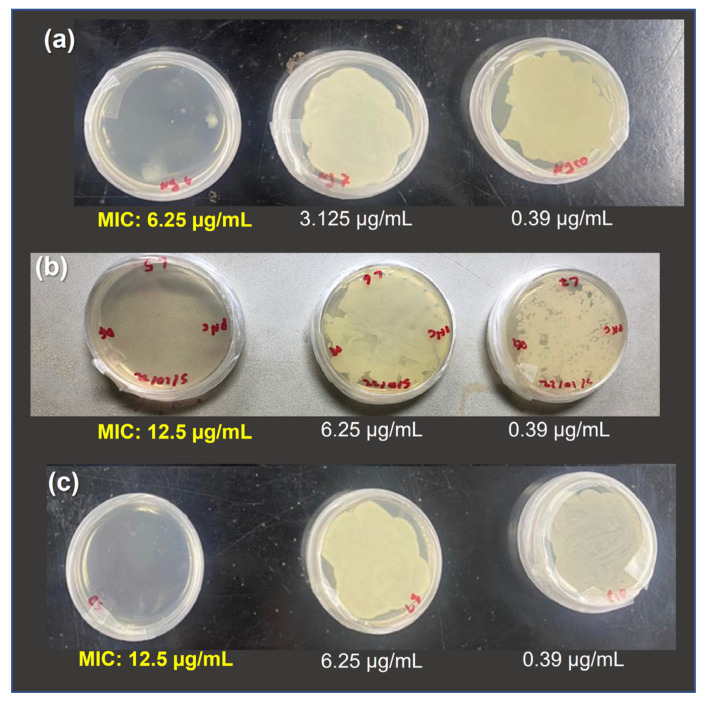
Minimum inhibitory concentration (MIC) of the synthesized hydrogels against *Vibrio harveyi*: (**a**) Chitosan_ZnO, (**b**) Chitosan_ZnO_Lc and (**c**) Chitosan_ZnO_Cx.

The lowest concentration of hydrogels at which there was no visible growth of *Vibrio harveyi* is shown in Figure 6. PB-loaded hydrogels were effective at 12.5 µg/mL meanwhile unloaded hydrogels were effective at a less concentrated dilution 6.25 ug/mL. These results showed that the antimicrobial activity of Chitosan_ZnO hybrid hydrogels is affected when loaded with PB.

### 3.5. Antimicrobial Activity of the Synthesized Hydrogels

**Figure 7:**
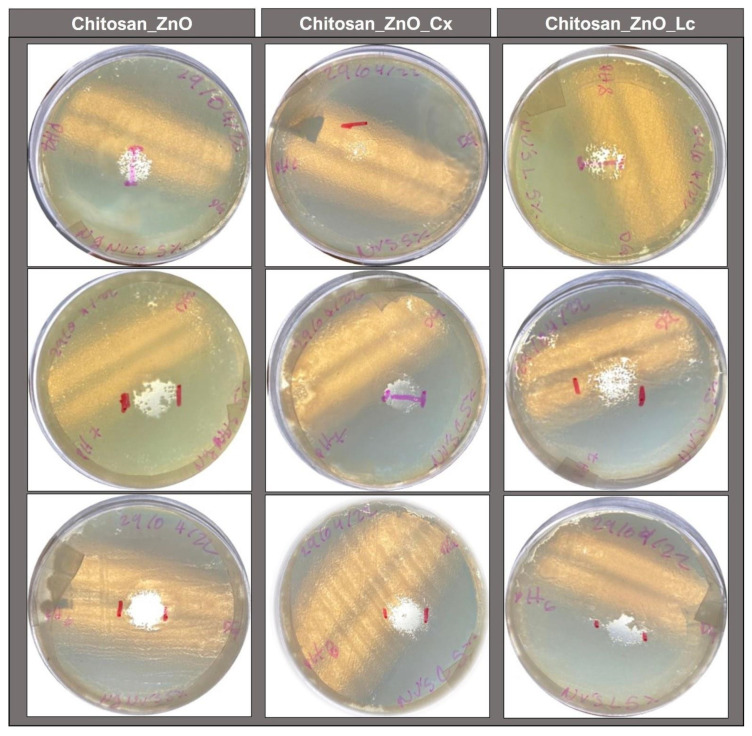
Inhibition zones (in triplicate) of the synthesized hydrogels against *Vibrio harveyi* (1 × 10^8^ CFU/mL).

Table 1 and Figure 7 display the inhibition halos produced by the chitosan synthesized hydrogels without loading and by those loaded with PBs against *Vibrio harveyi*. This shows that all synthesized hydrogels had inhibitory effects against these bacteria and that Chitosan_ZnO hydrogels had a powerful inhibitory effect by themselves even without PB loading. They also reveal that Chitosan_ZnO hydrogels loaded with Cx PB presented a small decrease in their antimicrobial activity. These results were not expected considering the antimicrobial properties of the PBs and the Chitosan_ZnO hybrid hydrogels. It should be noted that the pH of the culture medium was close to 7 and according to the swelling behavior of the synthesized hydrogels, in these conditions, the hydrogels allowed for the release of the encapsulated compound. However, the lower antimicrobial activity in the PB-loaded hydrogels may be due to the proton release by the chemical components of PBs, which could cause a pH decrease in the culture medium. As already determined for the synthesized hydrogels, low pH values favor the interaction between the components of the hydrogel, creating a stronger network that does not allow the PB to escape easily, inhibiting it from contributing to the antimicrobial activity of the hydrogel. Furthermore, it is also possible that due to this acidity, the hydrogel with Cx PB had less antimicrobial activity than the hydrogel with Lc. This could be caused by the hydroxyl group of phenol present in the compounds carvacrol and thymol (main components of Cx), which is more acidic than the hydroxyl group of cyclohexanol in menthol (a component of Lc) [68].

## 4. Conclusions

In this work, pH-responsive Chitosan AgCl co-doped ZnO hydrogels for the encapsulation of drugs and bioactive compounds were prepared. The synthesized porous polymeric network of Chitosan_ZnO hybrid hydrogels is formed via possible interactions between chitosan functional groups, TPP phosphoric ions and ZnO NRs that form a bigger network with more interactions and a better swelling behavior. The response of the hybrid hydrogel to a different pH solutions was evaluated through swelling behavior determinations and showed that low pH values cause the hydrogel polymeric network to expand and capture more of the aqueous solution. Meanwhile, neutral and basic pH values cause the hydrogel to shrink. These characteristics make the synthesized hydrogels suitable not only for the encapsulation and controlled release of drugs and bioactive compounds in aquaculture but also for other animals and humans. Chitosan_ZnO hybrid hydrogels showed great antimicrobial activity against *Vibrio harveyi*, even better than that of loaded PB hydrogels. Protons releasing from PB in loaded hydrogels probably cause a stronger encapsulation, inhibiting the release of the PBs. However, here, we provide evidence for the potential capacity of Chitosan_ZnO hybrid hydrogels for the preventive and curative treatment of diseases that impact aquaculture animal health and prevent drug resistance by bacteria. Further research is needed on the direct application and effect of hydrogel capsule administration on commercially important aquaculture organisms.

## Figures and Tables

**Table 1:** Hydrogel inhibition zone diameters of the synthesized hydrogels against *Vibrio harveyi* (1 × 10^8^ CFU/mL).

Hydrogel	Inhibition Zones Diameters (cm)
Chitosan_ZnO	1.53 ± 0.15
Chitosan_ZnO_Lc	1.43 ± 0.07
Chitosan_ZnO_Cx	0.83 ± 0.4

## Data Availability

The data presented in this study are available on request from the corresponding author.
